# Monoarthritis as the initial presentation of acute rheumatic fever in Iran: A single-center retrospective cross-sectional study 

**DOI:** 10.22088/cjim.15.2.328

**Published:** 2024

**Authors:** Vadood Javadi, Elham Mansourizadeh, Hassan Pourmoshtagh, Khosro Rahmani, Mehrnoosh Hassas Yeganeh

**Affiliations:** 1 Department of Pediatric Rheumatology, Shahid Beheshti University of Medical Sciences, Tehran, Iran; 2 Department of Pediatrics, Loghman-Hakim Hospital, Shahid Beheshti University of Medical Sciences, Tehran, Iran

**Keywords:** Acute rheumatic fever, Carditis, Arthritis, Monoarthritis, Children

## Abstract

**Background:**

In Iran, there is a lack of information and studies on acute rheumatic fever (ARF), a global health issue. The limited understanding of ARF's prevalence and primary clinical symptoms has led to confusion. This research investigates the characteristics of children aged 3-17 years who experience ARF with monoarthritis as their initial symptom.

**Methods:**

A retrospective evaluation of medical records of children diagnosed with ARF was conducted. The study aimed to determine the prevalence of monoarthritis as the first manifestation of ARF and its association with age, gender, family history, and cardiac involvement. Categorical variables were analyzed using the chi-square test with a significance level of < 0.05 and a confidence interval of 95%, using SPSS software (Version 23).

**Results:**

The study included 62 patients with ARF, comprising 41 (66.1%) boys with an average age of 8.48±3.27 years. Among these patients, 12 exhibited cardiac involvement according to the revised Jones criteria, with 5 clinical carditis and 7 cases of subclinical carditis. Monoarthritis was the initial symptom in seven patients (11.29%); five (71.4%) also had carditis. There was a significant association (*p<0.001*) between monoarthritis and carditis.

**Conclusion:**

The study concludes that monoarthritis may be an early sign of ARF in children and correlates significantly with cardiac involvement. However, more extensive research with more significant participant numbers is necessary to understand ARF in Iran comprehensively. A thorough cardiac examination is also crucial for patients with ARF and monoarthritis.

Acute rheumatic fever (ARF) is an autoimmune disease that occurs as a result of infection with Group A beta-hemolytic Streptococcal (GAS) bacteria in individuals with a genetic predisposition (1). ARF significantly impacts different tissues, such as the heart, joints, and central nervous system, and manifests several symptoms. Moreover, it is more common in school-age children globally (2). Recognizing the urgent need for enhanced surveillance, the 2018 World Health Assembly highlighted the importance of monitoring ARF cases (3).

In developing countries, risk factors for streptococcal infection, such as limited healthcare resources, poverty, overcrowding, malnutrition, and poor housing, contribute to an increased risk of acute rheumatic fever (4, 5). While the incidence of ARF has declined in developed countries in recent decades, it continues to pose a significant global health challenge despite the availability of effective treatment regimens (6).

The prevalence of ARF varies significantly across different regions, with the Middle East, Asia, Eastern Europe, and Australia reporting the highest incidence rates (10-350 cases per 100,000 people per year). In contrast, the United States and Western Europe exhibit the lowest incidence rates (0.5-3 cases per 100,000 people per year) (7). Iran, however, reports an incidence rate of 35 cases per 100,000 people (8). The primary manifestations of ARF include fever (occurring in over 90% of patients) and arthritis (occurring in 75% of patients) (2).

Nonetheless, a study by Dr. Jones et al. revealed that arthritis, carditis, chorea, subcutaneous nodules, and erythema marginatum constitute ARF's most frequently observed features (9). Cardiac involvement is the leading cause of mortality in ARF patients. Approximately 60% of untreated ARF patients are estimated to develop cardiac complications (10). Carditis, the most severe complication of ARF, is the most common acquired heart disease affecting all age groups and accounts for 50% of all cardiovascular disease cases and hospital admissions in developing countries (11). ARF cannot be diagnosed definitively by any clinical finding or lab test (12).

The modified Jones criteria, revised in 2015, diagnose ARF by categorizing the population into low-, medium-, and high-risk groups based on disease prevalence (2). In recent years, there has been a lack of understanding regarding the occurrence and clinical features of acute rheumatic fever (ARF) in Iran. Regrettably, no studies or publications have been conducted, resulting in limited available data on ARF's prevalence and primary clinical manifestations. The present study aimed to assess the specific attributes of children aged 3-17 years who are afflicted with ARF and display symptoms of monoarthritis.

## Methods

In this cross-sectional retrospective study, the medical records of patients diagnosed with ARF from 2017 to 2020 were reviewed. The patients aged between 3 and 17 years. They had been admitted to several academic hospitals in Tehran, Iran (Mofid Children’s Hospital, Loghman Hakim hospital, and Shohadaye Tajrish hospital). Monoarthritis as the initial presentation of ARF and, also, its relationship with age, sex, number of family members, and cardiac involvement were evaluated. In this study, monoarthritis was considered when it existed at least three days in one joint without the involvement of other joints or without the presence of immigrant arthritis. 

The presence of clinical carditis was suspected based on auscultation of a new pansystolic murmur. During the heart consultation, a pediatric heart specialist examined the long PR interval and used echocardiography to assess evidence of carditis, specifically involvement of the heart valves. Subclinical cases referred to patients who exhibited echocardiographic evidence of carditis but did not display clinical symptoms of congestive heart failure or a heart murmur. The diagnosis of ARF was carried out in line with revised Jones criteria(13). 

The quantitative results were given by mean, deviation, and percentage. Class variables were analyzed using the chi-square test. All data sets were significant at a level of less than 0.05 and at a confidence level of 95% using SPSS software (Version 23).

## Results

In the present study, 62 patients with ARF were studied, of whom there were 21 girls and 41 boys with an average age of 8.48±3.27 (table, supplementary). Twelve patients had cardiac involvement according to revised Jones criteria(13), five clinical carditides, and seven subclinical carditides, which were diagnosed by echocardiography. Five cases had carditis with bivalve involvement (mitral and aortic valves) and seven patients suffered isolated mitral valve disease (table 1). 

Moreover, seven (11.29%) out of the 62 patients admitted with acute rheumatic fever had monoarthritis. (table 2). 

Knee and ankle were equal the most commonly involved joints. Furthermore, the number of girls and boys were three and four cases, respectively, and there was no significant relationship between sex and monoarthritis. Among the patients with monoarthritis, five cases had heart involvement, two with clinical, and three with subclinical-type carditis. There was a significant association (p<0.001) between monoarthritis and carditis. In two patients, the mitral and aortic valves were involved, and isolated mitral valve involvement was observed in three cases (table 3). 

**Table 1:** Demographic characteristics and cardiac involvements of patients with ARF

**Variable**	**Frequency (%)**
**Sex**	
**Girl**	21 (33.9)
**Boy**	41 (66.1)
**Age**	
**Age**	8.48±3.27
**Family member number**	
**3**	14 (22.6)
**4**	32 (51.6)
**5**	16 (25.8)
**Cardiac Involvement**	
**Carditis**	12 (19.24)
**Clinical carditis**	5 (41.61)
**Sub clinical carditis**	7 (58.3)

**Table 2:** Clinical and laboratory findings of patients with monoarthritis

**No.**	**Gender**	**Age**	**Involved join**	**Other signs & symptoms**	**Echocardiographic findings**	**ESR (mm/1h)**	**CRP (mg/l)**	**WBC (/mm3)**	**ASO titer (Todd unit)**	**Throat culture**	**RF factor, HLA B27, HLA B5**
1	Male	6	Right hip	Fever & limping & history of pharyngitis about a month ago	normal	31	22	9800	400	Streptococci beta hemolytic group B	negative
2	Female	10	Right knee	Fever & pharyngitis 6 weeks ago	normal	35	21	5500	283	Normal flora	negative
3	Female	13	Spinal column	Pain in the joints of the lumbar spine & murmur in cardiac examination	Severe MR, moderate AR	55	147	14800	400	Normal flora	negative
4	Male	6	Right wrist	Pain &swelling of the right wrist joint	MR&AI	44	2+	7800	400	Normal flora	negative
5	Male	4	Right ankle	Fever &Pain &swelling of Right ankle	Mild AI& TR& MR	20	12	7500	300	Normal flora	negative
6	Female	7	Left knee	Skin rash & pain of Left knee	severe MR	20	negative	7200	600	Normal flora	negative
7	Male	11	Left ankle	Fever & limping& murmur in cardiac examination	MR&AI	40	20	7300	400	Normal flora	negative

**Table 3:** Characteristics of patients with ARF and monoarthritis

Variable	Frequency (%)	P-value
Sex		
Girl	3(42.89)	0.59
Boy	4(57.14)	
Age	8±3.46(3-17)	0.68
Family ember Number		
3	1(14.3)	0.68
4	3(42.9)	
5	3(42.9)	
Cardiac involvement		
Carditis	5(71.4)	<0.001
Clinical	2(40.0)	
Subclinical	3 (60.0)	
Joint involvement		
Ankle	2 (28.57)	
Knee	2 (28.57)	
Spinal column	1 (14.28)	
Wrist	1 (14.28)	
Hip	1 (14.28)	

## Discussion

In the realm of public health, ARF poses a significant challenge, particularly when it comes to children (14). The indigenous population typically exhibits monoarthritis and low-grade fever as the primary clinical manifestations of ARF, aligning with the conventional descriptions(11, 15). A retrospective cross-sectional study was conducted to evaluate the occurrence of monoarthritis as the initial indication of ARF in children who lived in Tehran, Iran, between 2017 and 2020.

The study included 62 patients diagnosed with ARF, consisting of 21 girls and 41 boys. The average age of children with ARF was 8.48±3.27 years. Notably, ARF exhibited a higher incidence in males, which is consistent with the findings of a previously published study on ARF, highlighting its greater prevalence among males than females (16).

 The reasons underlying the male predominance in ARF among the children remain unclear.

Our analysis disclosed that 11.29% of the 62 admitted ARF patients presented with monoarthritis as their initial symptom. Among these patients, the most common joints affected were the ankle and knee. The 2015 edition of the Jones Criteria has played a crucial role in resolving the variations observed in the clinical presentations of ARF among populations classified as low-risk, moderate-risk, and high-risk.

According to this criterion, monoarthritis is a significant clinical manifestation in moderate- to high-risk groups (13).

Numerous studies have examined the clinical presentation of ARF worldwide. The occurrence of monoarthritis in studies conducted by Bhutia et al. in India (12), Robazzi et al. in Brazil (17), Carapetis et al. in Australia (11), and Guler et al. in Turkey (10) was found to be 13%, 19.4%, 17%, and 16%, respectively. Additionally, these studies indicated that the knee joint was the most frequently affected, followed by the ankle joint. In contrast, Terreri et al. reported monoarthritis as the musculoskeletal manifestation in only 6% of ARF patients (18). Ralph et al. identified 36 suspected ARF cases, with only two presenting with monoarthritis (19). If left untreated, severe rheumatic valvular disease can lead to various complications, including atrial fibrillation, infective endocarditis (IE), heart failure, embolic events, and pulmonary hypertension (20). In our study, applying the revised Jones criteria, cardiac involvement was observed in 19.24% of all cases. Among these, 41.6% exhibited clinical carditis, while 58.4% had subclinical carditis. Bhutia et al.'s study indicated that 71.8% of cases had clinical carditis, and 14.6% had subclinical carditis (12). Harlan et al. discovered that all patients with monoarthritis displayed clinical evidence of carditis (21).

 Lilyasari et al.'s study, reported an exceptionally high prevalence of carditis, with 66.67% of patients presenting with carditis upon admission (20).

In general, anti-inflammatory medications such as naproxen and salicylates are commonly employed to manage the inflammatory process in ARF. However, using these drugs and other anti-inflammatory agents can give rise to atypical presentations of ARF (22). Furthermore, environmental factors, Group A Streptococcus (GAS) virulence, and individual susceptibility may contribute to unusual manifestations among ARF patients (15).

Our research findings indicate that monoarthritis can initially indicate acute rheumatic fever (ARF) in children, and there is a significant association between monoarthritis and cardiac involvement. The cardiac involvement observed in our study included both clinical and subclinical manifestations. However, it is essential to note that the number of patients included in our study was limited, which calls for further comprehensive investigations with a larger participant pool. 

A genetic predisposition may contribute to the coexistence of cardiac involvement and monoarthritis, and additional research is needed to explore this possibility. Furthermore, we strongly recommend thorough cardiac assessment for patients diagnosed with ARF and presenting with monoarthritis.
